# Therapeutic resistance in cancer: lncRNAs as modulators and targets for cancer therapy

**DOI:** 10.1016/j.gendis.2025.101990

**Published:** 2025-12-19

**Authors:** Ashiq Ali, Urooj Azmat, Aisha Khatoon, Bilal Murtaza, Kaynaat Akbar, Ziyi Ji, Urooj Irshad, Kinza Ishaque, Zhongjing Su

**Affiliations:** a School of Medical Sciences, Shandong Xiehe University, Jinan, Shandong, 250109, China; b Department of Histology and Embryology, Shantou University Medical College, Shantou, Guangdong 515041, China; c Department of Zoology, Wildlife and Fisheries, Faculty of Sciences, University of Agriculture, Faisalabad, Punjab 38040, Pakistan; d Department of Pathology, University of Agriculture, Faisalabad, Punjab 38040, Pakistan; e School of Bioengineering, Dalian University of Science and Technology, Dalian, Liaoning 114319, China; f Department of Zoology, Faculty of Sciences, Superior University, Lahore 51000, Pakistan

**Keywords:** Cancer treatment, Chemotherapy, Long non-coding RNAs, Radiation therapy, Therapeutic resistance

## Abstract

Therapeutic resistance continues to be a significant challenge in cancer treatment, often leading to tumor recurrence, metastasis, and limited therapeutic efficacy despite advancements in chemotherapy and radiotherapy. This resistance arises from complex molecular mechanisms that are influenced by various factors, including genetic alterations, epigenetic modifications, and cellular signaling. Recently, lncRNAs, a subclass of non-coding RNAs, have gained prominence for their essential role in driving cancer progression and mediating resistance to therapy. The resistance phenotype is shaped by lncRNAs through the regulation of key signaling pathways, DNA repair mechanisms, and cellular survival processes. For example, lncRNAs such as HOTAIR and MALAT1 are involved in the regulation of PI3K/AKT, EMT, and DNA repair pathways, and influence cell proliferation, apoptosis, and metastasis. These findings underscore the potential of lncRNAs as therapeutic targets. Targeting lncRNA-mediated pathways offers a promising approach to overcome resistance and enhance the efficacy of existing therapies. This review provides an in-depth analysis of the molecular mechanisms by which lncRNAs mediate resistance in various cancers, such as gastric cancer, lung cancer, breast cancer, and hepatocellular carcinoma. By focusing on the therapeutic potential of lncRNAs, we propose new avenues for overcoming resistance and improving treatment outcomes in cancer therapy.

## Introduction

In the ever-evolving battle against cancer, the discovery of long non-coding RNAs (lncRNAs) has opened a promising new frontier, offering transformative insights into tumor biology and potential therapeutic approaches. Recent advances in cancer research have shifted attention from traditional protein-coding genes to the vast and complex landscape of lncRNAs. These transcripts, longer than 200 nucleotides and lacking protein-coding potential, have emerged as critical regulators of diverse cellular processes, driving intense investigation into their roles in cancer development, progression, and treatment resistance.^1^ By exerting dual roles in promoting or suppressing tumor growth, lncRNAs have been identified as crucial regulators and hold significant promise as biomarkers and therapeutic targets in precision oncology.^2^ Cancer remains a formidable global health challenge, with treatments such as chemotherapy and radiotherapy significantly improving survival rates. However, resistance mechanisms, both innate and acquired, continue to hinder therapeutic success, often leading to tumor recurrence and metastasis. Emerging evidence highlights lncRNAs as key modulators of these resistance pathways, making the understanding and targeting of lncRNA-driven mechanisms a pressing priority in overcoming therapeutic resistance.^3^

Accounting for nearly 98% of the human genome, non-coding RNAs (ncRNAs) have emerged as vital regulators of cancer biology, controlling gene expression and critical cellular processes such as proliferation, apoptosis, migration, and invasion. Among these, lncRNAs have gained particular attention due to their diverse functional roles and complex regulatory mechanisms, alongside other important classes like micro RNAs (miRNAs) and circular RNAs (circRNAs).^4^ Depending on their specific targets, small non-coding RNAs known as miRNAs can act as oncogenes or tumor suppressors, and their dysregulation is associated with cancers such as breast cancer, lung cancer, and colorectal cancer. In contrast, lncRNAs, due to their greater length, interact with chromatin, proteins, and mRNAs to exert multifaceted control over gene expression. They play critical roles in tumorigenesis, metastasis, and notably, in mediating drug resistance, underscoring their significance in cancer progression and therapeutic response.^5^ While circRNAs, once considered splicing byproducts, are gaining recognition for their regulatory functions and potential as cancer biomarkers, lncRNAs remain at the forefront due to their diverse roles in modulating tumor biology and therapeutic resistance.

Beyond the extensively studied ncRNAs, other classes such as small nucleolar RNAs (snoRNAs), piwi-interacting RNAs (piRNAs), and small interfering RNAs (siRNAs) are increasingly recognized for their roles in tumor regulation. lncRNAs continue to be pivotal players in regulating cancer progression and therapeutic resistance through diverse and complex mechanisms.^6^ Furthermore, chemical modifications of lncRNAs, including methylation and pseudouridylation, critically impact their stability and function, highlighting the complex regulatory landscape of lncRNAs in cancer biology.^7^ A thorough understanding of these chemical modifications is essential to unravel how lncRNAs drive cancer progression and mediate resistance to therapy.^8^ This review examines the role of lncRNAs in mediating resistance to chemotherapy and radiotherapy across various cancers, elucidating the underlying molecular mechanisms and proposing targeted strategies to overcome resistance and improve therapeutic outcomes.

## Cellular localization and regulatory mechanisms of non-coding RNAs

In the intricate tapestry of gene regulation, miRNAs emerge as powerful orchestrators, deftly fine-tuning cellular processes and holding immense potential for therapeutic breakthroughs. Short RNA molecules that do not code for proteins, microRNAs (miRNAs) play crucial roles in gene regulation. They are initially transcribed as primary miRNAs (pri-miRNAs) in the nucleus, featuring a distinctive stem-loop structure. Following their transcription, pri-miRNAs are processed by the DROSHA–DGCR8 complex to produce precursor miRNAs (pre-miRNAs), which are then transported to the cytoplasm via XPO1.^9^ In the cytoplasm, the enzyme DICER1 cleaves pre-miRNAs, resulting in the formation of miRNA duplexes. One strand of this duplex is integrated into the miRNA-induced silencing complex (RISC), while the other strand is degraded.^10^ The RISC–miRNA complex selectively binds to target mRNAs through complementary base pairing, leading to either inhibition of protein synthesis or degradation of the mRNA. Beyond their classical roles, miRNAs also engage in non-traditional functions, such as activating Toll-like receptors (TLRs), binding to proteins outside of the AGO family, interacting with other ncRNAs through a process known as “sponging”, and influencing transcription.^11^

Although sharing a biogenesis pathway similar to mRNAs, lncRNAs possess complex three-dimensional structures that enable a diverse range of functions. They can be categorized based on their cellular localization: chromatin-bound (often involved in cis-regulatory functions), intranuclear (typically engaged in trans-regulatory roles), and intracytoplasmic (also performing trans-regulatory functions).^12^ Chromatin-bound lncRNAs influence transcription by facilitating chromosomal looping and modifying histones. Within the nucleus, lncRNAs can form paraspeckles and interact with nuclear proteins. In the cytoplasm, lncRNAs regulate gene expression post-transcriptionally by binding to messenger RNAs (mRNAs) as decoys, guides, or scaffolds, thereby affecting mRNA stability and translation. Additionally, lncRNAs can encode micropeptides, alter protein functions through binding, and interact with other ncRNAs, including miRNAs.^13^

Although arising from various biogenesis pathways, circRNAs are consistently generated through back-splicing events. These events can occur via protein dimerization, complementary sequences in adjacent introns, exon skipping, or the excision of intron lariats.^14^ Once back-spliced, circRNAs form continuous RNA loops that are transported to the cytoplasm, where they act as miRNA sponges, sequestering miRNAs and thus modulating the expression of target genes. circRNAs also serve as decoys for RNA-binding proteins, influencing gene expression and translation, and can act as platforms for protein interactions.^15^

**Figure 1:**
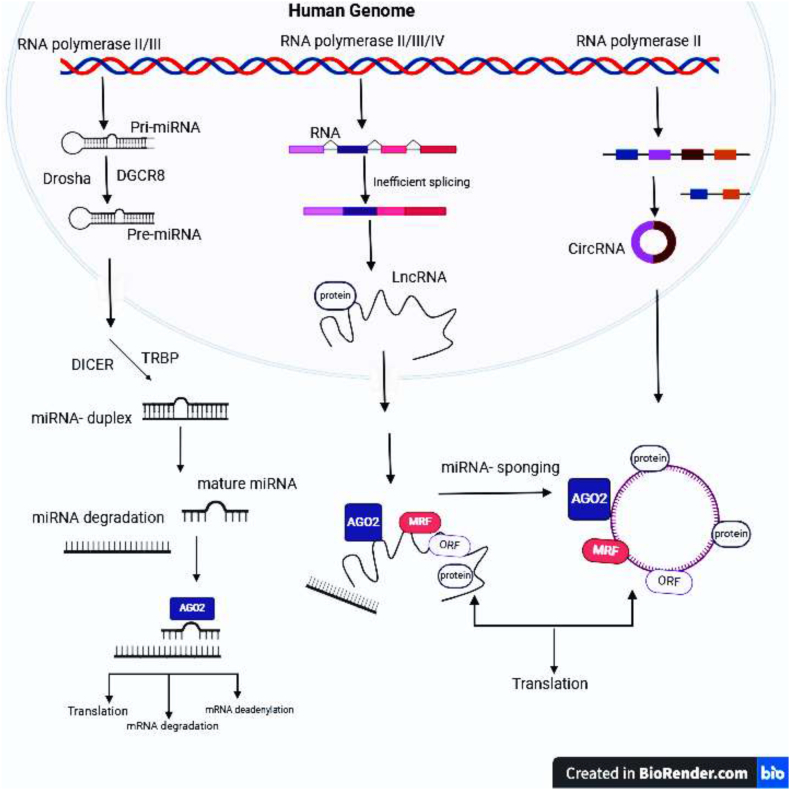
Biogenesis and functions of non-coding RNAs (ncRNAs). The miRNAs are transcribed as pri-miRNAs, processed by DROSHA and DGCR8 into pre-miRNAs, and exported to the cytoplasm, where DICER1 cleaves them into miRNA duplexes. One strand is incorporated into the RISC complex to inhibit target mRNA translation or induce degradation, while the other is degraded. miRNAs also perform atypical functions, such as activating Toll-like receptors, binding non-AGO proteins, and sponging other ncRNAs. The lncRNAs undergo capping, splicing, and acetylation, forming complex 3D structures that regulate gene expression. In the nucleus, they modulate chromatin, transcription, and nuclear protein interactions, while in the cytoplasm, they regulate mRNA, modulate protein functions, and interact with other ncRNAs, including miRNAs. circRNAs are formed via back-splicing and function as miRNA sponges, regulate RNA-binding proteins, and may translate into micropeptides, contributing to a regulatory ncRNA network.

Importantly, a dynamic exchange occurs among lncRNAs, miRNAs, and circRNAs through sponging mechanisms, resulting in intricate networks of ncRNA molecules. These networks are vital for regulating gene expression and various cellular processes.^16^ The synthesis and functions of these ncRNAs are illustrated in Figure 1.

## Specific role of lncRNAs in cancer therapy resistance

As key regulators of cellular processes and signaling cascades, lncRNAs play a pivotal role in driving resistance to cancer therapy. Their capacity to regulate gene expression, engage with microRNAs, and modify chromatin architecture designates them as essential contributors to treat resistance mechanisms.^17^ Numerous essential signaling pathways are facilitated by lncRNAs in this environment. Apoptotic pathways are often influenced by lncRNAs like MALAT1 and H19, which can obstruct programmed cell death and enhance cancer cell viability, therefore facilitating resistance to chemotherapy and radiotherapy.^6^ Likewise, the control of autophagy by lncRNAs such as PVT1 impacts drug sensitivity in non-small cell lung cancer. PVT1 regulates autophagy via interactions with microRNAs, influencing cisplatin resistance.

Besides apoptosis and autophagy, lncRNAs influence cell cycle regulation. MIR22HG indirectly promotes the synthesis of p21, a crucial inhibitor of cellular replication, hence affecting tumor cell proliferation and treatment efficacy. Moreover, lncRNAs like HOXA11-AS significantly contribute to the promotion of epithelial–mesenchymal transition (EMT), a mechanism that aids metastasis and increases resistance to targeted therapy.^18^ A different mechanism pertains to the regulation of drug efflux and transport. lncRNA NEAT1 can modulate the expression of drug transporter proteins, hence decreasing intracellular drug accumulation and impairing the efficacy of chemotherapy. This intricate control underscores the intricacy of lncRNA-mediated resistance.^19^

These pathways present advantageous therapeutic prospects. Methods like antisense oligonucleotides and siRNAs might selectively suppress lncRNAs associated with resistance mechanisms, potentially reinstating treatment sensitivity.^20^ Gene editing tools such as CRISPR/Cas9 offer enhanced capabilities to accurately target lncRNA genes. The efficacy of these medicines relies on the advancement of effective delivery mechanisms, such as nanoparticles, to guarantee targeted and efficient treatment. The development of resistance to cancer therapies is significantly influenced by lncRNAs, which modulate essential signaling pathways such as apoptosis, autophagy, cell cycle regulation, EMT, and drug efflux. These lncRNAs constitute a viable approach to improve therapy efficacy and surmount resistance in cancer patients.^21^

## Therapeutic strategies targeting lncRNA-mediated pathways

The pathways that are mediated by lncRNAs present promising prospects for the development of innovative cancer therapies, with various definitive methodologies arising from recent investigations. A viable strategy entails employing lncRNA inhibitors, specifically antisense oligonucleotides, which are short, synthetic nucleic acid strands engineered to selectively bind to and destroy target lncRNAs or obstruct their interactions.^22^ Antisense oligonucleotides targeting oncogenic lncRNAs such as MALAT1 have shown efficacy in diminishing tumor development and metastasis in preclinical models, underscoring their therapeutic promise. A potent method is RNA interference (RNAi), which employs tiny interfering RNAs (siRNAs) or short hairpin RNAs (shRNAs) to selectively inhibit lncRNA expression post-transcriptionally.^23^ RNAi-based therapeutics facilitate the exact regulation of lncRNAs implicated in drug resistance and tumor advancement. For instance, siRNA-mediated silencing of lncRNA PVT1 has been demonstrated to increase chemotherapy sensitivity in lung cancer cells by impairing autophagy pathways.^24^

The emergence of CRISPR/Cas9 genome-editing technology has enhanced the resources available for targeting lncRNAs. CRISPR-based methodologies can directly delete or change lncRNA genes in cancer cells, offering a precise and enduring mechanism to impair oncogenic lncRNA functions.^25^ This method facilitates the functional screening of lncRNAs to pinpoint critical resistance drivers, expediting the identification of therapeutic targets. Moreover, advancements in delivery systems such as lipid nanoparticles, exosomes, and conjugated polymers are essential for the clinical implementation of lncRNA-targeted therapeutics by guaranteeing stability, specificity, and effective cellular uptake.^26^ These efforts illustrate how targeting lncRNA-mediated pathways may transform cancer therapy by surmounting resistance, enhancing medication efficacy, and facilitating individualized treatment alternatives.

## Impact of lncRNAs on chemotherapy resistance in cancer

As the search for effective cancer therapies intensifies, lncRNAs are emerging as key players in the complex web of tumor biology, offering new insights into therapeutic resistance and disease progression. Exploring lncRNAs concerning malignancy has become increasingly popular in recent years. lncRNAs have been extensively studied for their involvement in several features of cancer, such as recurrence, metastasis, expansion, multiplication, propagation, immortality, angiogenesis, and therapeutic resistance.^27^ Comprehending the role of lncRNAs in therapeutic resistance is vital due to the intricate cellular contact networks. Various functions of lncRNAs have been identified in modulating resistance to different cancer therapies. Studies in this field have identified certain lncRNAs linked to treatment resistance in different cancer types. Cancer cell responses to chemotherapy, targeted therapy, and immunotherapy can be influenced by lncRNAs.^28^ Therapeutic resistance is impacted by lncRNAs through complex molecular pathways, often involving interactions with proteins and miRNAs that regulate essential cellular functions. Research on lncRNAs in cancer therapy resistance provides opportunities for creating specific therapeutic approaches. Researchers seek to uncover biomarkers for predicting medication response and discover therapeutic targets to overcome resistance by studying complex regulatory networks, including lncRNAs.^29^

## Dysregulation of lncRNAs in cancer

In the ever-evolving landscape of cancer research, lncRNAs are emerging as vital contributors to tumor biology, unveiling new dimensions in our understanding of malignancy and therapeutic targets. Numerous distinctive lncRNAs linked to cancer have also been detected.^30^ In contrast to small non-coding RNAs (sncRNAs), the roles of lncRNAs in cancer can significantly impact gene expression across epigenetic, transcriptional, and post-transcriptional dimensions.^2^ The Pan-Cancer Analysis of Whole Genomes (PCAWG) Consortium acknowledges the necessity for a repository providing information on lncRNAs with confirmed causative functions in cancer. The Cancer lncRNA Census (CLC) was created, consisting of 122 GENCODE-annotated lncRNA genes known for their well-established cancer-related roles. Among these genes, 77 are oncogenic, 35 act as tumor suppressors, and 10 exhibit both activities. Commonly observed lncRNAs in human cancers include HOTAIR, which interacts with polycomb repressive complex 2 (PRC2) to methylate and silence tumor suppressor genes; MALAT1, involved in alternative splicing; MEG3, a tumor suppressor regulating cell proliferation through TP53-dependent and independent pathways; and H19, activating cell survival pathways under stressful conditions.^31^ Table 1 provides a comprehensive overview of therapy resistance-associated lncRNAs across different cancers, their implicated pathways, and therapeutic targets.

**Table 1:** Summary of lncRNAs involved in therapy resistance.

lncRNA	Cancer type	Associated pathways	Therapeutic implications	Reference
MALAT1	Breast cancer, lung cancer	Apoptosis inhibition, epithelial–mesenchymal transition (EMT)	Antisense oligonucleotides to inhibit MALAT1 to reduce metastasis	^32^
HOTAIR	Breast cancer, thyroid cancer	EMT, chromatin remodeling	Targeting HOTAIR to suppress EMT and metastasis	^33^
PVT1	Non-small cell lung cancer	Autophagy regulation, apoptosis	RNAi-mediated knockdown to restore chemosensitivity	^34^
NEAT1	Lung cancer	Drug efflux regulation	Modulation of NEAT1 to overcome chemotherapy resistance	^35^
HOXA11-AS	Breast cancer	EMT, cell migration	Targeting to inhibit metastasis and therapy resistance	^36^
GAS5	Multiple cancers	Cell cycle arrest, apoptosis	Up-regulation as a tumor suppressor to sensitize therapy	^37^
UCA1	Bladder cancer, breast cancer	PI3K/AKT pathway activation	Targeting UCA1 to reduce resistance	^38^

## lncRNAs and chemoresistance in various types of cancers

In the intricate network of cancer biology, lncRNAs have emerged as pivotal players, influencing both tumor growth and the critical response to chemotherapy. Significant regulators of tumor growth and chemoresistance have emerged in the form of lncRNAs. They exert their influence through various mechanisms. Firstly, certain lncRNAs can alter the efflux of drugs from cancer cells, potentially affecting intracellular drug concentrations and chemotherapy efficacy. Secondly, lncRNAs are involved in modulating DNA damage repair mechanisms, thereby impacting the ability of cancer cells to respond to and repair damage caused by chemotherapeutic agents. Thirdly, they can influence apoptosis pathways, either promoting or inhibiting programmed cell death, which is targeted by many chemotherapeutic drugs.^39^ Additionally, lncRNAs may induce mutations in drug targets within cancer cells, leading to alterations in the effectiveness of targeted therapies. Notably, the majority of identified lncRNAs tend to promote chemoresistance, while only a few exhibit inhibitory effects, underscoring the complexity and diversity of their functions in cancer biology.

## lncRNAs in chemoresistance of gastric cancer

As one of the leading causes of cancer-related deaths worldwide, gastric cancer presents a formidable challenge, particularly in its ability to develop therapeutic resistance. Globally, gastric cancer is ranked as the second most prevalent cause of mortality and the fourth most prevalent malignancy. Addressing therapeutic resistance in gastric cancer presents a substantial problem that demands considerable effort. In cisplatin-resilient gastric melanoma cells, the lncRNA plasmacytoma variant translocation-1 (PVT-1) is significantly expressed.^40^ PVT1 demonstrates anti-apoptotic properties via increasing the expression of multidrug resistance protein 1 (ABCB1), multidrug resistance-associated protein 1 (MRP1, ABCC1), mammalian target of rapamycin (MTOR), and hypoxia-inducible factor 1 alpha (HIF-1α). This increase in activity leads to the development of resistance to chemotherapy.^41^ Elevated levels of lncRNA AK022798 result in increased ABCC1 levels and reduced expression of apoptotic-linked genes such as CASP3/8. This results in an antagonistic impact on the susceptibility of gastric cancer to cisplatin.^42^ Some lncRNAs have been shown to make gastric melanoma more sensitive to 5-fluorouracil. An instance is the lncRNA known as LEIGC, which functions as a tumor suppressor and is selectively down-regulated in gastric cancer. LEIGC effectively inhibits EMT, tumor development, and cell proliferation, leading to enhanced chemosensitivity in gastric cancer. The results highlight the several regulatory functions of lncRNAs in treatment resistance in gastric cancer, including involvement in proliferation, apoptosis, and EMT. This reinforces the idea that lncRNAs might act as prognostic indicators for treatment outcomes in gastric melanoma.^43^

## lncRNAs in chemoresistance of breast cancer

**Figure 2:**
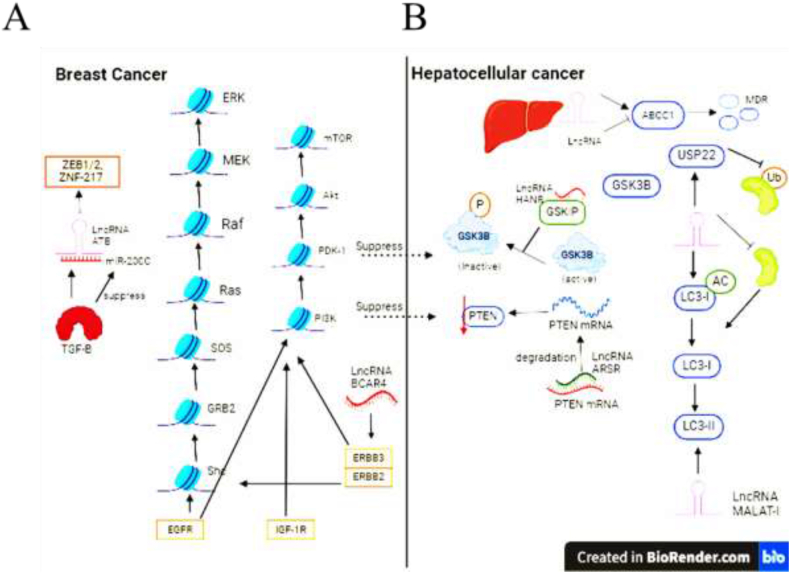
lncRNA-associated signaling pathways in breast cancer and hepatocellular carcinoma. **(A)** The lncRNAs influence oncogenic signaling pathways in breast cancer. Inhibition of miR-200c by lncRNA ATB leads to overexpression of the transcription factors ZEB1/2 and ZNF217, promoting epithelial–mesenchymal transition (EMT) and activating TGF-β signaling. This cascade activates EGFR and IGF-1R receptors, subsequently initiating the Ras/Raf/MEK/ESR1K and PI3K/AKT/MTOR pathways via adaptor proteins SHC1, GRB2, and SOS. lncRNA BCAR4 also facilitates the activation of ESR1BB2 and ESR1BB3, hence augmenting proliferation and survival signals in BC cells. **(B)** Modulation of critical oncogenic and tumor suppressor pathways by lncRNAs in hepatocellular carcinoma. lncRNA HANR suppresses the phosphorylation of GSK3B, preserving its active state by interaction with GSKIP. Active GSK3B inhibits PTEN expression at the transcriptional level, whereas lncRNA ARSR facilitates PTEN mRNA degradation, together resulting in the down-regulation of PTEN and the activation of downstream oncogenic pathways. lncRNA MALAT1 interacts with the autophagy regulator MAP1LC3B, facilitating the conversion from MAP1LC3B-I to MAP1LC3B-II via the deubiquitinase USP22, hence augmenting autophagic flux. The overexpression of ABCC1 mediated by lncRNA is associated with multidrug resistance (MDR) in hepatocellular carcinoma cells.

Breast cancer remains a daunting challenge as the most commonly diagnosed non-skin malignancy among women globally, necessitating a deeper understanding of its complex biology and treatment resistance.^44^ Breast cancer classification is commonly determined by gene expression patterns, leading to subtypes such as luminal A and B, basal-like, HESR12-enriched, and normal breast-like groups.^45^ For persons with estrogen receptor (ESR1), the primary approach to management revolves around regulating estrogen stimulation and employing anti-hormonal therapies such as estrogen deprivation and tamoxifen (TAM). However, unexpected circumstances frequently occur in patients, resulting in the emergence of significant chemoresistance. lncRNA HOTAIR shows a significant increase in TAM-resistant breast cancer. The elevated expression of HOTAIR is linked to accelerated disease advancement and severity, indicating its potential as a therapeutic target for those unresponsive to TAM therapy.^46^ Different lncRNA-related pathways in breast cancer have been depicted in Figure 2A.

Breast cancer anti-estrogen resistance 4 (BCAR4) is involved in resistance to TAM in breast cancer. BCAR4 acts as a strong oncogene that reduces the sensitivity of breast cancer to estrogenic stimulation and anti-hormone treatment, partly by affecting the ESR1BB2/ESR1BB3 pathway. Individuals with high levels of BCAR4 are more likely to develop resistance to chemotherapy.^47^ CCAT2 is involved in promoting cell growth and inhibiting cell death in TAM-resistant cells, contributing to colon cancer progression. Targeting the depletion of CCAT2 is a new strategy to combat resistance in patients.^48^ An instance is the lncRNA activated by transforming growth factor beta (lnc-ATB), which shows increased expression in breast cancer that is resistant to trastuzumab. lnc-ATB is involved in inducing chemoresistance, facilitating tumor progression, and resulting in a worse prognosis. It achieves this by competitively sequestering miR-200c, consequently activating zinc finger E-box binding homeobox 1 (ZEB1) and ZNF-217.^49^ The studies offer new perspectives on the role of lncRNAs in breast cancer treatment. Several lncRNAs enhance cancer aggressiveness and spread by activating subsequent ways. It is essential to discover new lncRNAs that might be beneficial for patients with chemoresistant breast cancer. This continuous investigation shows potential for creating innovative approaches to address medication resistance in breast cancer and enhance treatment results.^50^

## lncRNAs in chemoresistance of hepatocellular carcinoma

Hepatocellular carcinoma (HCC) stands as a formidable global health threat, demanding urgent advances in therapeutic strategies due to its aggressive nature and high mortality rate. HCC is a major international health issue, being one of the deadliest tumors globally. HCC makes up 90% of all liver cancer cases. Individuals diagnosed with late-stage HCC frequently undergo chemotherapy treatment, which may involve widely used drugs like doxorubicin, sorafenib, 5-fluorouracil, and platinum-based medications.^51^ One significant obstacle in treating HCC is the emergence of multidrug resistance (MDR) despite the presence of chemotherapeutic choices. This syndrome presents a major challenge in clinical treatment. Patients who develop MDR infections are more prone to showing a suboptimal reaction to chemotherapy, resulting in negative consequences like metastasis, recurrence, and an overall grim outlook.^52^ Research on overcoming multi-drug resistance in HCC is now a focus of study. Comprehending the molecular processes of MDR and investigating the role of different components, such as lncRNAs, is essential for developing improved treatment strategies. Enhanced understanding of the intricacies of HCC advancement and resistance mechanisms is crucial for creating precise and individualized therapy approaches for individuals with this aggressive liver cancer. HANR directly interacts with GSKIP, resulting in reduced glycogen synthase kinase 3 beta (GSK3B) phosphorylation. This relationship affects glycogen metabolism and cell proliferation, leading to decreased susceptibility to chemotherapy. On the other hand, inhibiting HANR expression hinders the growth of HCC in experimental and live conditions. Moreover, this inhibition triggers programmed cell death and increases susceptibility to the chemotherapeutic medication.^53^

The expression of ATP-binding cassette subfamily C member 1 (ABCC1) can be up-regulated by lncRNA NR2F1-AS1 through its modulation of miR-363. ABCC1, an integral part of the ATP-binding cassette (ABC) transporter superfamily, is commonly associated with MDR. The link between NR2F1-AS1 and resistance to oxaliplatin has been confirmed.^54^^,^^55^ Some lncRNAs contribute to developing resistance to certain medicines. The extended ncRNA HULC, markedly elevated in HCC, diminishes the efficacy of oxaliplatin, 5-fluorouracil, and pirarubicin by stimulating autophagy via the inhibition of the Sirt1 protein. Different lncRNA-related pathways in HCC have been depicted in Figure 2B.

lncRNA HULC may boost the production of peptidase 22 ubiquitin-specific while reducing the ubiquitin-dependent breakdown of the Sirt1 protein. This impact is accomplished by breaking the linked polyubiquitin chain of Sirt1.^56^ MALAT1 is markedly increased in HCC that is resistant to 5-fluorouracil, doxorubicin, and mitomycin, indicating its association with metastasis. Suppressing MALAT1 can restore chemosensitivity, leading to reduced MAP1LC3B-II levels and enhanced 5-fluorouracil-induced apoptosis. In general, numerous lncRNAs appear to play a part in diverse drug resistance mechanisms in HCC by impacting downstream pathways. The methods involve suppressing phosphorylation, intervening in metabolism, generating MDR expression, and offering new perspectives for enhancing treatment techniques.^57^

## lncRNAs in chemoresistance of lung cancer

**Figure 3:**
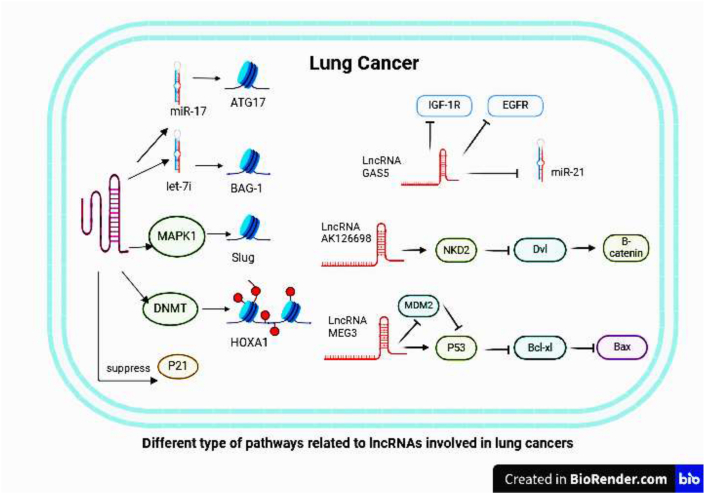
Various molecular mechanisms via which lncRNAs modulate oncogenic and tumor-suppressive pathways in lung cancer. Signaling pathways are modulated by lncRNAs through their interactions with microRNAs, transcription factors, and protein regulators. lncRNA ATG17 is governed by miR-17, whereas let-7i influences BAG-1, resulting in the activation of MAPK1 and the subsequent expression of the transcription factor SNAI2, so facilitating epithelial–mesenchymal transition (EMT). The control of DNA methyltransferases (DNMTs) by lncRNA results in the methylation and overexpression of HOXA1, which inhibits P21, thereby promoting tumor cell proliferation. lncRNA GASS5 reduces IGF-1R/EGFR-mediated miR-21 production, whereas lncRNA AK126698 lowers NKD2, hence boosting Dvl activation and β-catenin signaling, which contributes to tumor proliferation. lncRNA MEG3 interacts with MDM2, thereby stabilizing TP53, which then regulates BCL2L1 and BAX to facilitate apoptosis. These lncRNA-driven regulatory networks collectively serve vital roles in regulating cell proliferation, survival, apoptosis, and metastasis in lung cancer.

Multidrug resistance in lung cancer poses a formidable barrier to effective chemotherapy, demanding innovative solutions to overcome therapeutic failure. Platinum-based chemotherapy is the main therapy available to lung cancer patients. Regrettably, a notable proportion of individuals have been documented to acquire multidrug resistance. Cisplatin-resistant cells show differential expression of many lncRNAs.^58^ HOTAIR can lead to medication resistance, although its sensitivity can be partially restored by silencing it using siRNA. P21 has been recognized as a downstream mediator of HOTAIR. An improved amount of p21 may effectively counteract HOTAIR-induced resistance to cisplatin in laboratory studies. Lowering HOTAIR levels leads to decreased HOXA1 methylation, which enhances the effectiveness of chemotherapy in small cell lung cancer (SCLC).^59^ The anti-oncogene lncRNA maternally expressed gene 3 (MEG3) is expressed at lower levels in cisplatin-resilient cells. Exogenously introducing MEG3 can control the countenance of TP53 and BCL2L1, leading to the restoration of cisplatin resistance in a laboratory setting.^60^ Different lncRNA-related pathways in lung cancer have been shown in Figure 3.

Additionally, lincAK126698 has been demonstrated to hinder cisplatin-induced cell death by decreasing the expression of bare cuticle homolog 2 and enhancing β-catenin transcription.^61^ Likewise, lncRNA-XIST reduces the effectiveness of cisplatin in cancer cells by affecting the lncRNA-XIST/miR-17/autophagy regulatory pathway and the let-7i/BAG-1 pathway, leading to reduced cell death and enhanced cell growth.^62^ It has been recorded that the lncRNA BLACAT1 is increased in cisplatin-resistant non-small cell lung cancer cells. BLACAT1 enhances autophagy and resistance to chemotherapy.^63^ The lncRNA, nicotinamide nucleotide transhydrogenase antisense RNA1 (lncRNA NNT-AS1), is significantly present in cisplatin-resistant non-small cell lung cancer tissues and cells. lncRNAs also influence the effectiveness of gefitinib. Increased expression of GAS5 has been demonstrated to hinder tumor growth by directly targeting the insulin-like growth factor 1 receptor (IGF-1R). Alternatively, blocking linc00635–001 while undergoing gefitinib treatment might lead to decreased AKT levels, ultimately overcoming drug resistance.^64^ Dysregulation of several lncRNAs in lung cancer can impact the resistance or susceptibility to chemotherapy by affecting apoptosis and autophagy pathways.

## lncRNAs in cancer radioresistance

Radioresistance remains a critical challenge in cancer therapy, as certain tumors leverage cellular mechanisms to evade the destructive effects of radiation. Radiation therapy is becoming an essential technique in treating cancer patients. Some malignancies are more resistant to radiation therapy due to biological complexity, heterogeneities, and the existence of cancer stem cells (CSCs), leading to the reappearance of malignancy, proliferation, and unfavorable prognoses. Research has shown that lncRNAs can influence radioresistance by affecting DNA damage repair, apoptosis, controlling EMT, and regulating malignant primary cell activities.^65^ Table 2 summarizes key lncRNAs involved in drug radioresistance, associated pathology, and resistance mechanisms.

**Table 2:** Summary of lncRNAs involved in drug radioresistance.

Pathology	lncRNA	Drug	Mechanism of resistance	Reference
Breast cancer	MALAT1	Doxorubicin	Inhibition of apoptosis and promotion of epithelial–mesenchymal transition	^66^
Lung cancer	PVT1	Cisplatin	Autophagy regulation and apoptosis inhibition	^67^
Colorectal cancer	HOTAIR	5-Fluorouracil	Epithelial–mesenchymal transition induction and chromatin remodeling	^59^
Gastric cancer	UCA1	Cisplatin	Activation of PI3K/AKT pathway	^68^
Non-small cell lung cancer	NEAT1	Gefitinib	Up-regulation of drug efflux transporters	^69^
Glioma	TUG1	Temozolomide	DNA damage repair modulation	^70^
Esophageal cancer	CCAT1	Radiotherapy	Enhanced DNA repair and radioresistance	^71^
Cervical cancer	GAS5	Cisplatin	Modulation of apoptosis pathways	^72^

## lncRNAs in radioresistance of cervical cancer

**Figure 4:**
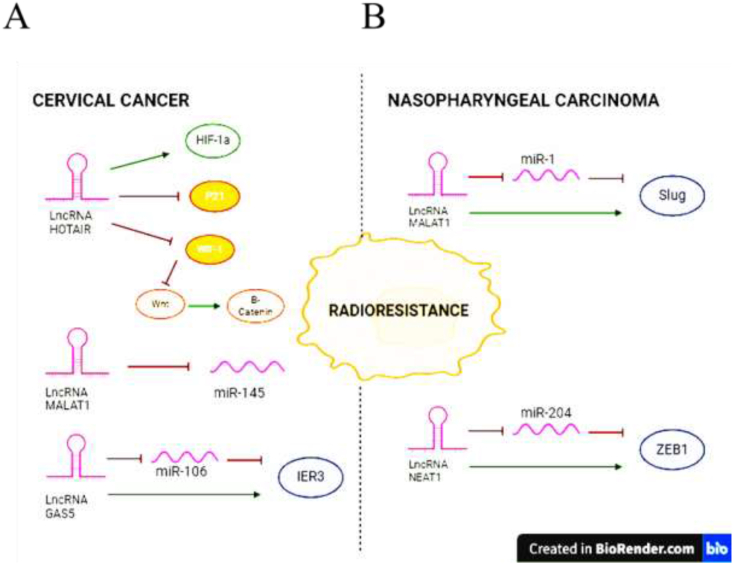
lncRNA-associated pathways mediating radioresistance in cervical cancer and nasopharyngeal carcinoma. **(A)** lncRNAs facilitate radioresistance in cervical cancer via many molecular processes. lncRNA HOTAIR enhances HIF1A expression and diminishes P21 levels, facilitating cell survival and resistance to therapy. Moreover, HOTAIR suppresses WIF-1, thus increasing the WNT/β-catenin signaling pathway, which is associated with increased proliferation and resistance to radiation. lncRNA MALAT1 inhibits miR-145, hence enhancing radioresistance. lncRNA GAS5 similarly inhibits miR-106 expression, resulting in the overexpression of IESR13, a gene responsive to stress that promotes cell survival after radiation exposure. **(B)** Specific lncRNAs facilitate radioresistance in nasopharyngeal cancer by regulating variables associated with epithelial–mesenchymal transition. lncRNA MALAT1 functions by sequestering miR-1, resulting in the overexpression of SNAI2, a transcription factor that facilitates epithelial–mesenchymal transition and confers treatment resistance. lncRNA NEAT1 inhibits miR-204, leading to elevated ZEB1 expression, an additional component driving epithelial–mesenchymal transition linked to radioresistant phenotypes. The lncRNA–miRNA–transcription factor axes collectively highlight the crucial function of ncRNAs in modulating tumor radioresistance pathways.

Therapeutic resistance in cervical cancer, particularly to radiation, is a formidable barrier to treatment success, driven in large part by intricate lncRNA-regulated pathways. Cervical cancer is a prevalent gynecological cancer globally. The incidence and fatality rates have increased throughout the years. Individuals suffering from cervical malignancy are often subjected to lots of operations, radiation, or chemotherapy. Nevertheless, therapeutic resistance frequently leads to a grim prognosis and has a chance of endurance of 40%–50%.^73^ HOTAIR interacts with the WNT signaling way to stimulate autophagy, promote EMT, boost cell proliferation, and suppress apoptosis in response to radiation. Additionally, HOTAIR suppresses p21 expression and increases HIF1A levels in irradiated cervical cancer cells, leading to the development of radio-resistance. Cell metabolism regulation plays a critical role in the emergence of resistance to radiation. lncRNA UCA1, along with the enzyme hexokinase-2 (HK2), has been demonstrated to enhance glycolysis in radioresistant tumor cells, impacting the sensitivity to radiation. MALAT1 reduces the G2 checkpoint and constrains programmed cell death in exposed malignant cells by functioning as a sponge for miR-145.^74^ Unlike the previously stated lncRNAs, the increased expression of lncRNA GAS5 has been shown to improve the responsiveness of malignant cells to radiation. The GAS5 up-regulates immediate early response-3 (IESR1-3) expression by blocking microRNA-106b. The GAS5/IESR13/microRNA-106b axis enhances the susceptibility of malignant cells to radiation. These discoveries illustrate that various lncRNAs affect the efficacy of radiation, with many contributing to radioresistance by promoting cell proliferation, modulating metabolism, and influencing cell cycle arrest.^75^ Different lncRNA-related pathways in cervical cancer have been shown in Figure 4A.

## lncRNAs in radioresistance of hepatocellular carcinoma

**Figure 5:**
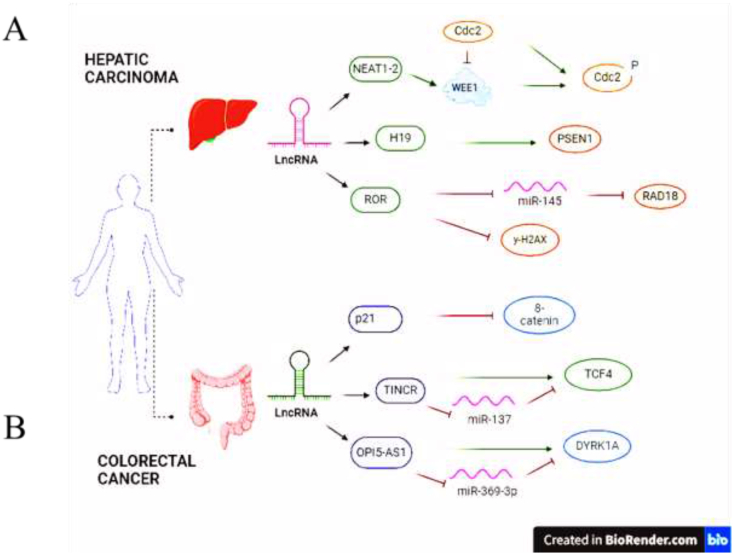
lncRNA-associated pathways in hepatic carcinoma and colorectal cancer. **(A)** Numerous lncRNAs modulate essential molecular pathways that drive tumor growth and therapeutic resistance in hepatocellular carcinoma. lncRNA NEAT1-2 facilitates tumor proliferation by suppressing WEE1, resulting in unregulated activation of CDK1 and advancement of the cell cycle. lncRNA H19 promotes oncogenesis by up-regulating PSEN1, whereas lncRNA ROR down-regulates miR-145, resulting in RAD18 overexpression and increased DNA repair activity. Moreover, ROR inhibits γ-H2AX, thereby diminishing DNA damage signaling and facilitating radio and chemoresistance in hepatocellular carcinoma. **(B)** In colorectal cancer, lncRNAs regulate oncogenic and resistance pathways. lncRNA TINCR suppresses p21, thereby alleviating the repression of β-catenin signaling and facilitating tumor proliferation via TCF4 activation. Moreover, TINCR diminishes miR-137 expression, hence augmenting tumorigenic pathways. lncRNA OPI5-AS1 operates as a competitive endogenous RNA (ceRNA) by sequestering miR-369-3p, resulting in the overexpression of DYRK1A, a kinase linked to tumor survival and resistance to therapy. These lncRNA-mediated pathways collectively regulate cell cycle regulation, DNA repair, and oncogenic signaling in colorectal cancer.

In HCC, radioresistance remains a major challenge, with specific lncRNAs orchestrating DNA repair mechanisms that undermine the effectiveness of radiotherapy. Radiotherapy is a potential treatment for HCC, with lncRNAs playing a role in regulating DNA repair to decrease radioresistance, as depicted in Figure 5A. Typically, hereditary damage is fixed during the G1 checkpoint. But when the G1-S checkpoint is lacking, cancer cells tend to use the G2 arrest to repair disturbed heredity. WEE1 kinase, a specific kind of tyrosine kinase, aids in the restoration of altered heredity by halting the G2 arrest. Increased expression of lncRNA NEAT1_2 and WEE1, together with decreased levels of miR-101-3p, can encourage malignancy when exposed to radiation, leading to decreased effectiveness of treatment.^76^ Linc-ROR, recognized as a lncRNA controller of programming, has also been observed to be up-regulated in radioresistant HCC cell lines. Down-regulation of Linc-ROR has been demonstrated to improve treatment effectiveness in laboratory settings and living organisms by suppressing the ability to repair DNA. It acts as a competitive endogenous RNA (ceRNA) for miR-145, regulating RAD18 expression to enhance DNA repair and resistance to radiation. lncRNA TP73-AS1, overly prominent in radioresistant malignant tissues, was identified as a promoter of radiation resistance in hepatic melanoma by activating the PTEN/AKT signaling way. Down-regulation of TP-73-AS1 has been demonstrated to inhibit cell growth, promote cell death, and ultimately improve the sensitivity to radiation therapy. The lncRNA H19/microRNA-193a-3p axis has been shown to promote malignancy by targeting presenilin 1 (PSEN1), a key part of γ-secretase, and inducing therapeutic tolerance. Overall, these investigations show that lncRNAs have a substantial impact on radio-resistance in HCC by affecting DNA damage repair processes. This element shows potential for future study and possible uses in clinical trials.^77^

## lncRNAs in radioresistance of colorectal cancer

In colorectal cancer (CRC), the battle against radioresistance is intensified by lncRNAs, which manipulate cell survival and stem cell dynamics, undermining treatment efficacy. CRC is the major mortality contributor among cancerous diseases. While many patients with CRC respond well to radiotherapy, some individuals acquire radioresistance, which can result in poor prognosis.^78^ lncRNAs play a role in radiation resistance in CRC through apoptosis and CSCs, as shown in Figure 5B. For instance, the increased levels of terminal differentiation-induced non-coding RNA (TINCR) have been confirmed to contribute to radioresistance in CRC cells. Suppressing TINCR hinders the activity of transcription factor 4 (TCF4) by influencing the countenance of microRNA-137. TCF4 is linked to the activity of CSCs. The finding of lncRNA-p21 has been found to reduce β-catenin signaling transduction, leading to decreased survival, regeneration, and glycolysis of CSCs in laboratory conditions. This ultimately increases the sensitivity of CRC cells to radiation therapy.^79^

Similarly, lncRNA OIP5-AS1 can increase the sensitivity of tumor cells to radiation by suppressing the production of miR-369-3p. This hindrance leads to heightened levels of dual-specificity tyrosine phosphorylation-regulated kinase 1A (DYRK1A), a gene located downstream of microRNA-369-3p.^80^ Both lncRNA OIP5-AS1 and DYRK1A overexpression enhance programmed cell death in malignant cells after radiation exposure. Moreover, the up-regulation of lncRNA UCA1 in CRC is associated with therapeutic resistance. Down-regulation of UCA1 inhibits colony formation, proliferation, and EMT while simultaneously triggering apoptosis and the mitotic G2 checkpoint. HOTAIR's connection to caspase-mediated cell death directive after treatment with radiation indicates its potential as a therapeutic target.^81^ The research shows that lncRNAs can influence apoptosis and CSC-like features, potentially helping to combat radioresistance in CRC patients.

## lncRNAs in radioresistance of nasopharyngeal carcinoma

In nasopharyngeal carcinoma, radioresistance fueled by lncRNA-driven mechanisms poses a significant obstacle to effective therapy, highlighting critical targets for therapeutic intervention. Numerous processes are being recognized as factors that contribute to resistance to radiation in nasopharyngeal carcinoma, as shown in Figure 4B. MALAT1 can enhance the function of CSCs and promote resistance to radiation. Moreover, lncRNA NEAT1 has been shown to increase ZEB1 expression by capturing miR-204, thereby having an impact on EMT and the tumor's resistance to radiotherapy. Furthermore, research has shown that lncRNA PVT1 is notably increased in nasopharyngeal melanoma cells. When PVT1 is suppressed, it leads to heightened susceptibility to radiation, marked by reduced cell growth and higher cell death. This indicates that PVT1 shows potential as a beneficial target.^82^ lncRNAs are involved in controlling malignant primary cells, apoptosis, and EMT, which leads to the development of radioresistance in nasopharyngeal carcinoma.

## Regulatory functions of lncRNA modifiers in carcinogenesis

RNA modifications like m^6^A are emerging as pivotal regulators in cancer therapy resistance, transforming lncRNA functions to bolster tumor survival against treatment pressures. Specific modernizers, particularly m^6^A, are essential for modifying lncRNAs in tumor cells, which leads to therapeutic resistance. m^6^A-related changes can improve the functionality of lncRNAs by affecting RNA processing, transport, and stability. An example is the documented m^6^A-induced alteration of the lncRNA RP11, which enhances the advancement and spread of CRC via increasing ZEB1 post-translationally.^83^ The research emphasizes the complex regulatory functions of RNA modifications, particularly m^6^A, in influencing the functional characteristics of lncRNAs and their effects on cancer biology and response to therapy. Elevated levels of lncRNA RP11 have been linked to decreased effectiveness of cisplatin and erlotinib, resulting in resistance to chemotherapy in tumor cells. The m^6^A modification is highly concentrated in lncRNA FAM225A, leading to enhanced RNA stability. The observations emphasize the various ways in which lncRNAs, specifically RP11 and FAM225A, together with their alterations like m^6^A, can impact chemotherapy resistance in cancer cells. Comprehending these relationships offers crucial insights for creating ways to combat resistance and enhance treatment results. lncRNA FAM225A functions as a ceRNA by binding to miR-590-3p and miR-1275. This interaction results in the up-regulation of the downstream gene integrin-β3 (ITG-β3) and triggers the FAK/PI3K/AKT signaling cascade.^84^ These molecular processes collectively increase the growth and aggressiveness of nasopharyngeal carcinoma, leading to resistance to treatment.

The ceRNA mechanism demonstrates how lncRNAs such as FAM225A can regulate gene expression and signaling pathways, impacting the cellular response to cancer therapy. lncRNA MALAT1 is widely recognized for its involvement in malignancies. Studies have shown that the methyl transferase-like protein 16 (METTL-16), which is an N6-methyladenosine encoder, can preferentially attach to the 3′ end of the metastasis-associated lung adenocarcinoma transcript 1 (MALAT1) is protected by a triple-helical structure.^85^ This structure is AU-rich and highly conserved. The interaction between METTL-16 and MALAT-1 may be linked to the cancer-causing properties of MALAT-1. Moreover, m^6^A mutation can cause a specific alteration in the configuration of MALAT-1, increasing its affinity for heterogeneous nuclear ribonucleoprotein C (HNRNPC) and resulting in the creation of an m^6^A switch.^86^ The results emphasize the complex regulatory processes that involve MALAT1 and m^6^A alteration, revealing their significance in cancer biology. m^6^A mutation can cause the degradation of lncRNAs, which can result in treatment resistance in malignant cells. lncRNA growth arrest specific transcript 5 (GAS5) has been confirmed a prevent malignancy development in various experiments by binding directly to microRNA-21 and thereby increasing the susceptibility to chemotherapy. GAS5 affects the response to cisplatin in cervical carcinoma by controlling AKT phosphorylation. The results highlight the significance of m^6^A alteration in regulating the stability and function of lncRNAs, which affects their contribution to chemotherapy resistance in cancer cells. Cervical cancer resistance to cisplatin is controlled by lncRNA GAS5, which modulates AKT phosphorylation. The m^6^A mutation triggers the breakdown of GAS5, with the help of m^6^A reader proteins YTHDF2 and YTHDF3. This mechanism enhances resistance to therapy and is linked to a negative prognosis. Pseudouridine (ψ) has been associated with specific tumor-related lncRNAs, such as MALAT1 and RN7SK, in addition to m^6^A.^87^ Additional research is needed to understand the processes and roles of pseudouridine and supplementary modifiers in the complex interaction between lncRNAs and resistance to treatments.

## Challenges in translating lncRNA-based therapies to clinical practice

Although targeting lncRNAs in cancer holds significant therapeutic promise, numerous difficulties must be addressed to convert these techniques into successful clinical interventions. A significant obstacle is the creation of secure and effective delivery mechanisms. lncRNAs are sizable, frequently intricate molecules that necessitate particular delivery systems such as lipid nanoparticles, viral vectors, or exosomes to guarantee their arrival at target cells in adequate amounts without destruction.^88^ Attaining tissue-specific delivery to reduce systemic exposure continues to be a considerable challenge. Another problem pertains to off-target effects, as lncRNAs may engage in many interactions inside the cell, occasionally influencing genes or pathways unrelated to the intended target. This intricacy increases the likelihood of unforeseen outcomes, such as toxicity or disruption of normal cellular functioning. Developing precise inhibitors and conducting comprehensive preclinical evaluations are crucial to reduce these dangers.^89^

Moreover, the variability of tumors and the fluctuating expression of lncRNA present further difficulties. Patient variability and intra-tumoral heterogeneity can affect treatment responses, limiting the identification of universal lncRNA targets. Tailored strategies and extensive biomarker analysis will be essential for optimizing therapy efficacy.^90^ The long-term safety and efficacy of lncRNA-targeted therapies have yet to be conclusively determined. As new medicines transition from the laboratory to clinical trials, thorough assessment of their efficacy, pharmacokinetics, and possible immunogenicity will be essential. Confronting these issues necessitates a multidisciplinary approach that amalgamates molecular biology, nanotechnology, and clinical oncology to fully exploit the potential of lncRNA-based cancer therapeutics.^91^

## Future directions in lncRNA research and cancer therapy

The functions of lncRNAs in cancer have unveiled their significant impact on therapeutic resistance, paving the way for more precise and targeted treatment approaches in oncology. lncRNAs and their regulatory networks play vital roles not only in cancer initiation and progression but also in driving resistance to multiple therapies. By influencing key downstream genes and signaling pathways, lncRNAs contribute to the complexity of treatment failure, highlighting their potential as both biomarkers and targets for overcoming resistance in future cancer therapies.^92^ lncRNAs influence critical cellular processes such as apoptosis, proliferation, autophagy, and tumor cell migration. They also promote traits associated with CSCs and regulate EMT, both are key factors in therapy resistance and metastasis. These insights highlight the exciting potential of targeting lncRNAs and their regulatory networks in cancer treatment. Overall, investigating the roles of lncRNAs, especially their contribution to treatment resistance, is a rapidly evolving and promising field. As our knowledge expands, it holds great promise for the development of more precise and personalized therapies that can overcome resistance and improve patient outcomes.^93^

Advances in biotechnology, including high-throughput sequencing, bioinformatics analysis, genome editing, animal models, and medicinal chemistry, have greatly expanded our ability to study lncRNAs and their regulators. These cutting-edge tools are deepening our understanding of how lncRNAs contribute to cancer biology and therapy resistance, opening new possibilities for innovative diagnostic methods, targeted treatments, and preventive strategies in oncology.^94^ Distinctive expression profiles of lncRNAs and their associated regulators, detected in the serum of cancer patients, may serve as valuable biomarkers for tumor staging and monitoring disease progression. These functional RNA molecules play crucial roles in cancer development by acting as either oncogenic drivers or tumor suppressors. Importantly, lncRNAs are key players in the emergence of drug resistance, influencing treatment outcomes. Their tissue-specific expression patterns and notable stability in body fluids like serum make them especially promising candidates for non-invasive cancer diagnostics and monitoring.^95^ The remarkable stability of lncRNAs, along with their unique expression profiles, underscores their potential as powerful cancer biomarkers. Because they can accurately mirror tumor progression and therapy resistance, lncRNAs are emerging as valuable targets for research and clinical use in cancer diagnosis, prognosis, and treatment monitoring. Notably, specific lncRNAs as well as miRNAs have been directly associated with treatment response and patient outcomes, highlighting their promise in guiding personalized cancer care.^96^ Their capacity to modulate resistance or sensitivity to chemotherapy and radiation therapy highlights the critical clinical importance of lncRNAs in influencing cancer treatment outcomes.

A fundamental cellular mechanism, RNA interference (RNAi), naturally regulates gene expression by selectively silencing target genes at the post-transcriptional level. This process is driven by small double-stranded RNA molecules that are complementary to the mRNA of the gene to be silenced. Inside the cell, these RNA molecules are processed and integrated into the RNA-induced silencing complex (RISC), which guides the degradation or repression of the target mRNA. Leveraging RNAi technology offers promising avenues for targeting lncRNAs involved in cancer progression and therapy resistance.^96^ The RNA-induced silencing complex (RISC) directs the RNAi machinery to target mRNAs, leading to their degradation or translational repression, thereby silencing specific gene expression. RNAi is integral to many biological processes, including development, antiviral defense, and genome stability maintenance. Its precision and post-transcriptional regulation make RNAi a valuable approach for investigating gene function and developing targeted therapies. For example, antisense oligonucleotides targeting lncRNA MALAT1 have demonstrated the ability to promote differentiation and reduce metastasis in breast cancer models, underscoring the therapeutic potential of modulating lncRNAs in cancer treatment.^97^ Additionally, the use of antisense oligonucleotides to inhibit lncRNA MALAT1 has been shown to suppress metastasis in lung melanoma xenograft models. Increasing evidence indicates that dysregulation of lncRNAs is widespread in cancer, where they critically influence processes such as CSC maintenance, metastasis, and therapy resistance. This dysregulation highlights the promising potential of lncRNAs as therapeutic targets for improving cancer treatment outcomes.

Acting as either oncogenes or tumor suppressors, lncRNAs modulate key cellular functions such as proliferation, apoptosis, migration, and invasion.^98^ In CSCs, lncRNAs play crucial roles in maintaining self-renewal, guiding differentiation, and initiating tumors. Dysregulation of lncRNAs is frequently linked to increased metastatic capacity and the emergence of drug resistance in cancer cells. Identifying lncRNAs involved in cancer development has paved the way for novel therapeutic approaches that harness lncRNA-based strategies. Approaches such as lncRNA mimics or inhibitors to restore or suppress their function show great potential for effectively targeting key cancer pathways.^17^ Overall, the dysregulation of lncRNAs in cancer and their key roles in tumor progression underscore their potential as promising therapeutic targets for more precise and personalized cancer treatments. Emerging strategies include the use of nanoparticles for delivery, techniques to modulate lncRNA expression, and oncolytic adenovirus-based approaches aimed at effectively targeting lncRNA-mediated pathways.

Recent advances in delivery technologies have propelled multiple clinical trials focused on RNA-guided precision medicine. While miRNAs currently dominate this landscape, with several progressing to late-stage trials such as miR-31-3p and miR-31-5p in colorectal cancer and miR-21 and miR-200 in oral melanoma. As delivery methods improve, lncRNA-targeted therapies hold considerable promise for future clinical development and personalized cancer treatment.^99^ Clinical studies are increasingly incorporating lncRNAs and circRNAs, exemplified by MALAT1, which is being investigated in breast and lung cancers. Additionally, siRNAs, often delivered via lipid nanoparticles, have advanced into clinical trials. Notable siRNA-based therapies include DCR-MYC, targeting the MYC oncogene to inhibit cell proliferation in hepatocellular carcinoma, and Atu-027, designed to suppress PKN3 and limit cell motility in metastatic pancreatic adenocarcinoma. These developments underscore the potential of lncRNAs and other RNA-based molecules as valuable biomarkers for monitoring therapy response and as targets to personalize cancer treatment strategies.^100^ Despite these promising advances, significant challenges remain before lncRNA-based therapies can be widely adopted in clinical practice. The vast diversity in length, structure, and function of lncRNAs, coupled with their intricate interactions with regulatory molecules across different cancer types, complicates the identification of precise and effective therapeutic targets. Addressing these challenges requires deeper insights gained through comprehensive genomic and functional analyses in both basic research and translational studies, which are essential for advancing lncRNA-based precision medicine.^101^ Moreover, developing effective delivery systems with strong target specificity remains a significant challenge, even when ideal receptors are identified. The heterogeneity of the tumor microenvironment further complicates the efficient transport and uptake of lncRNA-based therapeutics. Key obstacles include limited transfection efficiency, off-target effects, and the inherently short half-life of RNA molecules due to instability and degradation. Overcoming these barriers is critical for the successful clinical application of lncRNA-targeted therapies.^102^ Additionally, much of the current research remains confined to specific tumor types or particular treatment modalities.^103^

To effectively translate these findings into clinical practice, it is essential to expand research to encompass a broader range of tumor types and therapeutic approaches. Following the identification of promising lncRNA targets and reliable delivery systems, rigorous clinical trials are needed to assess patient responses to lncRNA-based therapies. Such studies are crucial for the understanding of long-term effects and uncovering potential side effects that may have been previously overlooked. Despite current challenges, the dual roles of lncRNAs as oncogenic drivers or tumor suppressors present exceptional opportunities for developing innovative treatments. These strategies hold significant promise for overcoming therapeutic resistance and improving survival outcomes for patients facing refractory cancers.^104^

## Conclusion

In conclusion, lncRNAs and their modifiers hold transformative potential as therapeutic targets for overcoming drug resistance and enhancing cancer treatment outcomes. Their unique roles in regulating critical cancer processes, including apoptosis, proliferation, and EMT, as well as CSC-like properties, highlight their importance in driving therapeutic resistance. With advances in biotechnologies and delivery mechanisms, ncRNA-based therapies are entering clinical trials, particularly targeting lncRNAs in diverse cancer types. Despite ongoing challenges related to precise targeting, bioavailability, and tumor microenvironment complexities, innovations in delivery systems such as nanoparticles and receptor-specific carriers promise to improve therapeutic precision. Expanding research across a broader range of tumors and treatment methods will be key to translating these discoveries into effective, personalized treatments, ultimately paving the way for lncRNA-based therapies to redefine cancer management.

## CRediT authorship contribution statement

**Ashiq Ali:** Writing – review & editing, Writing – original draft, Visualization, Validation, Conceptualization. **Urooj Azmat:** Writing – original draft. **Aisha Khatoon:** Conceptualization. **Bilal Murtaza:** Validation. **Kaynaat Akbar:** Writing – original draft. **Ziyi Ji:** Visualization, Validation. **Urooj Irshad:** Software. **Kinza Ishaque:** Validation, Software. **Zhongjing Su:** Writing – review & editing, Writing – original draft, Visualization, Validation, Supervision, Conceptualization.

## Data availability

The data will be available on request to the corresponding author.

## Conflict of interests

The authors declared no conflict of interests.
